# Depressive Symptoms and Their Association With Quality of Life in Older Adults With Cataracts: A National Survey in China

**DOI:** 10.31083/AP45683

**Published:** 2025-08-11

**Authors:** Zi-Mu Chen, Meng-Yi Chen, Qinge Zhang, Yuan Feng, Zhaohui Su, Teris Cheung, Gang Wang, Chee H. Ng, Yu-Tao Xiang

**Affiliations:** ^1^Unit of Psychiatry, Department of Public Health and Medicinal Administration, & Institute of Translational Medicine, University of Macau, Taipa, Macao, China; ^2^Centre for Cognitive and Brain Sciences, University of Macau, Taipa, Macao, China; ^3^Beijing Key Laboratory of Mental Disorders, National Clinical Research Center for Mental Disorders & National Center for Mental Disorders, Beijing Anding Hospital, Capital Medical University, 100088 Beijing, China; ^4^School of Public Health, Southeast University, 210008 Nanjing, Jiangsu, China; ^5^School of Nursing, Hong Kong Polytechnic University, Hong Kong, China; ^6^Department of Psychiatry, The Melbourne Clinic and St Vincent’s Hospital, University of Melbourne, Richmond, VIC 3121, Australia

**Keywords:** cataracts, older adults, depression, quality of life, network analysis

## Abstract

**Background:**

Depression is common among older adults with cataracts and is associated with significant functional impairment. However, the complex interrelationships among different depression symptoms are often overlooked by conventional mood disorders research based on total scores of depression measures. This study examined the interrelationships between different depressive symptoms and quality of life (QoL) in older adults with cataracts based on a national survey. By analyzing the key depressive symptoms related to QoL in this vulnerable population, the study aimed to identify potential critical treatment targets.

**Methods:**

In this study, the 10-item Center for Epidemiologic Studies Short Depression Scale and the World Health Organization Quality of Life-brief version were used to measure depressive symptoms and QoL respectively. In the network analysis, Expected Influence was used to identify the central symptoms, and a flow network model was used to examine the symptoms that directly affected QoL.

**Results:**

A total of 1683 participants were included in the analysis. Economic status was the only identified risk factor for depression in older adults with cataracts. The most central symptoms in the depression network were “Feeling blue”, “Everything was an effort”, and “Inability to get going”. The flow network indicated that QoL had the strongest direct connections with “Unhappiness”, “Sleep disturbances” and “Feeling blue”.

**Conclusions:**

Depression was found to be common among older adults with cataracts. To mitigate the negative impact of depression on QoL, psychosocial interventions targeting the most central symptoms and those directly related to QoL should be prioritized.

## Main Points

1. Economic status was identified as a risk factor for depression in older adults with cataracts.

2. The most influential depressive symptoms among older adults with cataracts were “Feeling blue”, “Everything was an effort” and “Inability to get going”.

3. The depressive symptoms that were most directly correlated with QoL were “Unhappiness”, “Sleep was restless” and “Feeling blue”.

## 1. Introduction

Depression is one of the most common and important causes of disability globally [1]. It is associated with a range of negative health outcomes, particularly in older adults with chronic physical diseases, including marked functional and cognitive impairment resulting in substantial burden on the individual, their family, and society at large [2]. With an aging population in China, certain eye conditions, such as cataracts, are emerging as a risk factor for depression, particularly among older adults [3, 4]. Having such comorbidity aggravates the disease burden and contributes to reduced quality of life (QoL).

Cataracts are characterized by the loss of lens transparency due to lens opacification. The condition predominantly manifests as age-related cataracts in older adults [5]. Cataracts are among the leading causes of clinically significant vision loss worldwide in adults aged 50 years and older, affecting approximately 33.6 million individuals globally in 2020 (15.2 million cases [95% uncertainty interval (UI) 12.7–18.0]) [6]. Previous research has consistently demonstrated a close link between cataracts in older adults and an elevated risk of depression [7, 8], which is primarily related to visual impairment that limit physical activity and restrict social engagement [9, 10]. Therefore, to reduce the negative psychosocial impact of vision impairment on older adults with cataracts, understanding the pattern of depression among this population is important.

Quality of life is a widely used health outcome, and several QoL domains, such as physical and psychological health, social relationships, and environmental factors, are usually measured in research [11]. Previous research has found that effective interventions for depression are associated with an improvement in QoL [12], which not only reflects the relationship between depression and QoL, but also indicate that adequate management of depression can enhance life satisfaction and overall well-being in older adults. Although it is widely known that depression has a significant impact on QoL, to date, no studies have examined such impact among older adults with cataracts. Furthermore, the interrelationships between different depressive symptoms and QoL in this population have not been explored.

Conventionally, studies have only explored the relationship between cataracts and depression at a syndrome level, using aggregate scores from depression assessments. However, depression consists of a range of different symptoms, involving mood (i.e., depressed mood, psychic anxiety), cognitive (i.e., concentration difficulties), somatic (i.e., lack of energy) and sleep domains (i.e., early, middle, and late insomnia), with each having different neuro-psychological mechanisms [13]. In recent years, network analysis has offered new insights into the psychopathology and interrelationships among various psychiatric symptoms [14], which can be calculated mathematically and presented visually. The most influential (central) symptoms are identified using several centrality measures in the network model [15], which can either activate other symptoms or are activated by them, thereby maintaining the symptom network as a whole [16]. To date, no studies on the inter-relationships between depressive symptoms in older adults with cataracts have been published.

Therefore, our study aimed to investigate the prevalence, correlates and network structure of depression in relation to QoL in older adults with cataracts, utilizing data from a national survey in China.

## 2. Methods

### 2.1 Study Design and Population

**Fig. 1.**
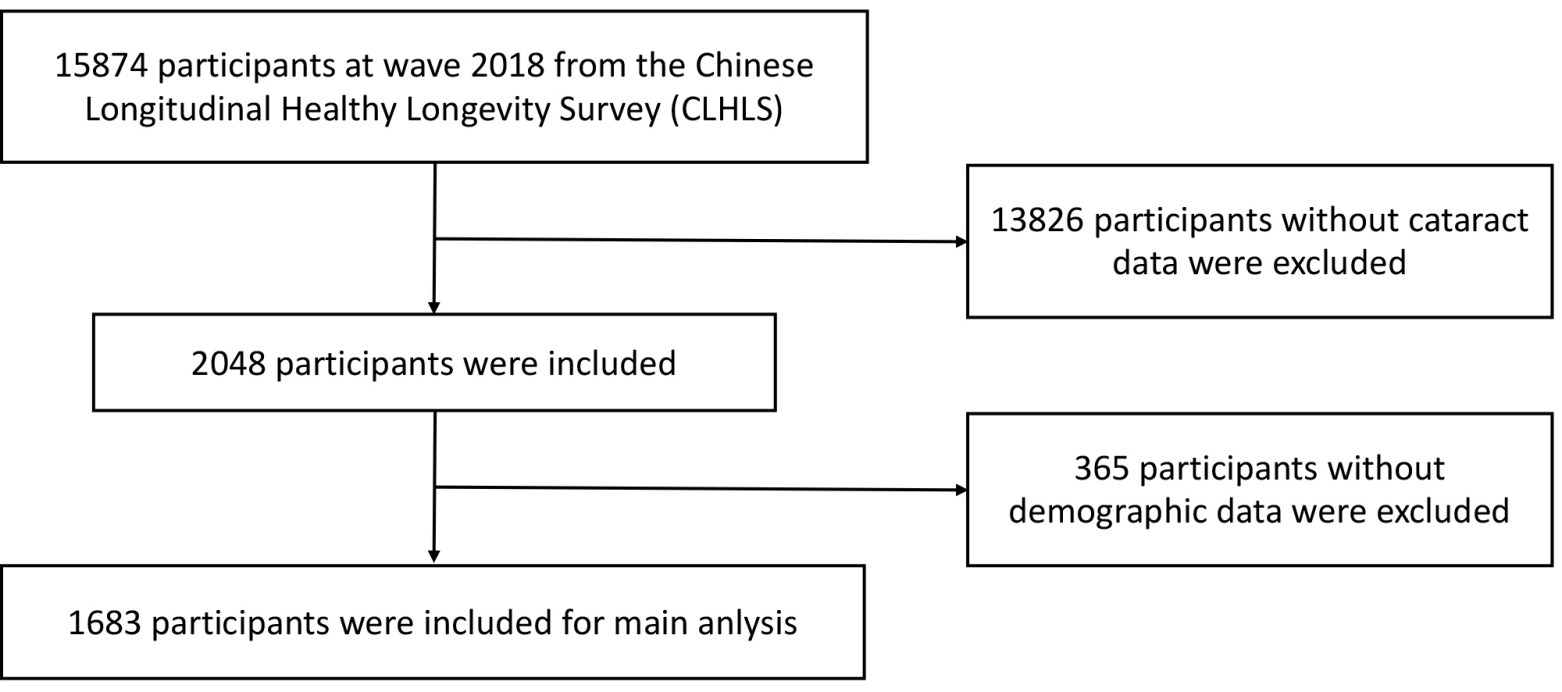
**Flowchart for the selection of the analysed study sample from the Chinese Longitudinal Healthy Longevity Survey (CLHLS)**.

The study was based on the Chinese Longitudinal Healthy Longevity Survey (CLHLS), which evaluated the health status and QoL of the older adults aged 65 and older via face-to-face home-based interviews, in randomly selected 23 out of the 31 Chinese provinces from 1998 to 2018 [17, 18]. The study mainly focused on the determinants of healthy aging and mortality in the oldest-old, by analysing aspects such as physical and mental health, socioeconomic characteristics, lifestyle, family dynamics and demographic details of older adults [17, 18]. The data for this cross-sectional analysis were derived from the 2018 wave of the CLHLS, which was released in 2020. All the 15,874 participants aged 65 and older from the CLHLS 2018 wave were included in the study. Following previous research [19, 20], having cataracts was determined using the following standardized question: “Are you suffering from cataracts?” To be eligible, participants without cataract records or complete records of demographic characteristics were excluded, leaving 1683 participants for the present study (see Fig. 1).

### 2.2 Measurement of Demographic Characteristics

Socio-demographic information was collected to examine the risk factors for depression and QoL among older adults with cataracts. The data captured included age, gender, education level, marital status, living status, economic status and current smoking and drinking behaviors.

### 2.3 Assessment of Depressive Symptoms 

Severity of depression was evaluated using the validated Chinese version of the 10-item Center for Epidemiologic Studies Short Depression Scale (CESD-10), which has been validated in terms of its reliability and consistency across different age groups (Cronbach α = 0.78) [21]. The CESD-10 encompasses the following affective states: ‘Feeling Bothered’, ‘Feeling blue’, ‘Hopelessness’, ‘Feeling fearful’, ‘Unhappiness’, and ‘Loneliness’. Additionally, it comprises three cognitive states (‘Concentration difficulties’, ‘Everything was an effort’, and ‘Inability to get going’) and one sleep state (‘Sleep disturbances’) [13]. Participants rated the frequency of these symptoms over the preceding week on a four-point scale ranging from 0 (‘rarely or none of the time’) to 3 (‘most or all of the time’). The total score of CESD-10 ranges from 0 to 30, and the cut-off value of 10 has been validated in older adult populations to reliably identify partcipants as having depression.

### 2.4 Definition of Global QoL

The first two components of the World Health Organization Quality of Life scale – Brief version (WHOQOL-BREF) were extracted to provide a measurement of global QoL [11]. The first two components of the WHOQOL-BREF consisted of overall perception of QoL (item1), and satisfaction with general health facet (item2). Psychometric evaluation in Chinese older adults showed satisfactory properties (Cronbach’s α = 0.86) [22]. Both items were scored on a 5-point scale ranging from 1 to 5, where higher scores indicated better QoL.

### 2.5 Statistical Analysis

Baseline characteristics between participants with depression and those without depression were compared using independent sample Mann-Whitney U tests or Pearson Chi-square tests, as appropriate. Analysis of covariance (ANCOVA) was applied to examine the independent relationship between QoL and depression, after adjusting for significant variables identified in the initial analyses. A two tailed *p*-value of *p* < 0.05 was considered as statistically significant.

### 2.6 Network Structure

The package qgraph version 1.9.8 was utilized to visualize the network [23]. The network structure was computed using Extended Bayesian Information Criterion (EBIC) combined with the least absolute shrinkage and selection operator (LASSO) [15]. Nodes in the network represent various depressive symptoms or QoL, while each edge between two nodes indicates their association after accounting for the other nodes in the model. Stronger interactions are represented by thicker, more saturated edges. Edges in green denote positive relationships, whereas edges in red indicate negative ones. Mixed graphical models via nodewise regression was used to calculate the prediction ratio of a node based on all its neighboring nodes, which serves as an essential factor in assessing the practical significance of specific edges [24].

Node centrality is crucial for understanding individual node importance within a network model [15]. Centrality index of Expected Influence (EI), which quantifies the influence of a node with both positive and negative edges within a network, can predict how changes in one node relate to changes in others [25]. In the model, high EI nodes are more significant than those with low EI in terms of understanding mental disorder development, persistence, and remission within network theory contexts [25]. The packages bootnet version 1.5.6 was used to compute the above indices [15].

In addition, the “flow” function in the package qgraph version 1.9.8 was employed to designate QoL as a source node and identify direct and indirect connections to other nodes, thus maximizing predictive pathways while accounting for all variables in the model. Node-specific predictive betweenness, was calculated to determine the shortest predictive pathways between QoL and other nodes. Given that betweenness is typically an unstable centrality metric, the variability extent was calculated by both nonparametric and case-drop bootstraps [15].

### 2.7 Network Stability

The packages bootnet version 1.5.6 was employed to assess robustness and replicability of network [14, 15].

The correlation stability-coefficients (CS-coefficient) for EI and strength were calculated to investigate network stability by ensuring a minimum correlation of 0.7 with 95% probability between original and subset samples after the maximum drop proportions of cases were removed from the original sample. The correlation coefficent should not fall below 0.25 and preferably be above 0.5 [15]. Edge weights along with 95% confidence interval (CI) were computed using the non-parametric bootstrapping method, where narrower CIs suggest a more reliable network structure [15]. Additionally, the stability of EI and edge weights was further examined using bootstrapped difference tests [15]. Moreover, the stability analysis of average node-specific predictive betweenness was performed, which provided additional insights under case-dropping conditions.

All the statistical analyses were conducted using R program version 4.3.1 (Foundation for Statistical Computing, Vienna, Austria) [26]. The multivariate imputation by chained equations were carried out using package mice version 3.16.0 [27].

## 3. Results

### 3.1 Demographic Characteristics

In total, 1683 older adults with cataracts were included, after excluding 14,191 individuals due to incomplete data on cataracts or essential demographic characteristics. Of the included participants, 642 (38.1%) were male, and the average age was 87.4 (standard deviation (SD) = 10.9) years. The prevalence of depression (CESD-10 total score ≥10) in older adults with cataracts was 16.5% (95% CI: 14.5–18.3%) in this study sample.

### 3.2 Potential Covariates of Depression

Table 1 shows the differences in the baseline demographic characteristics between participants with and without depression. Older adults with cataracts and concurrent depression were more likely to be female (*p* = 0.020), and have a lower education level (*p* = 0.022) and a lower economic level (*p* < 0.001).

**Table 1.** **Demographic and clinical characteristics of the study sample**.

		Total		Depression		No Depression		Univariate
		(n = 1683)		(n = 277)		(n = 1406)		analyses
		N	%	N	%	N	%	*p* value
Male Gender		642	38.1	88	31.8	554	39.4	**0.020**
Junior education level		468	27.8	61	22.0	407	28.9	**0.022**
Married		632	37.6	92	33.2	540	38.4	0.118
Living with others		1336	79.4	215	77.6	1121	79.7	0.476
Perceived economic level								< **0.001**
	Poor	156	9.3	61	22.0	95	6.8	
	Fair	1130	67.1	186	67.1	944	67.1	
	Good	397	23.6	30	10.8	367	26.1	
Current smoking		458	27.2	69	24.9	389	27.7	0.385
Current drinking		368	21.9	67	24.2	301	21.4	0.345
		Mean	SD	Mean	SD	Mean	SD	*p* value
Age (years)		87.4	10.9	87.2	10.7	87.4	10.9	0.751
Global QoL		7.2	1.4	6.1	1.4	7.4	1.4	< **0.001**

Notes: Bolded values: <0.05; SD, standard deviation; QoL, Quality of Life.

### 3.3 Association Between QoL and Depression

Older adults with cataracts and concurrent depression exhibited lower QoL scores (F = 130.1, *p* < 0.001), compared to those without depression, after adjusting for covariates. The logistic regression analysis showed that a higher economic level (odds ratio (OR) = 0.309, *p* < 0.001; OR = 0.13, *p* < 0.001) was significantly linked to a reduced risk of depression (Table 2).

**Table 2.** **Independent correlates of depressive symptoms among older adults with cataract (n = 1683)**.

Variables		Depression		
		OR	95% CI	*p*
Male Gender		0.799	0.590–1.082	0.146
Junior education level		0.997	0.711–1.398	0.986
Married		0.864	0.638–1.170	0.344
Perceived economic level				
	Poor	1.000		
	Fair	0.309	0.215–0.444	< **0.001**
	Good	0.130	0.079–0.215	< **0.001**

Notes: The *p* value of this model <0.001. Bolded value: <0.05; Abbreviation: CI, confidential interval; OR, odds ratio.

### 3.4 Network Structure of Depression With QoL

**Fig. 2.**
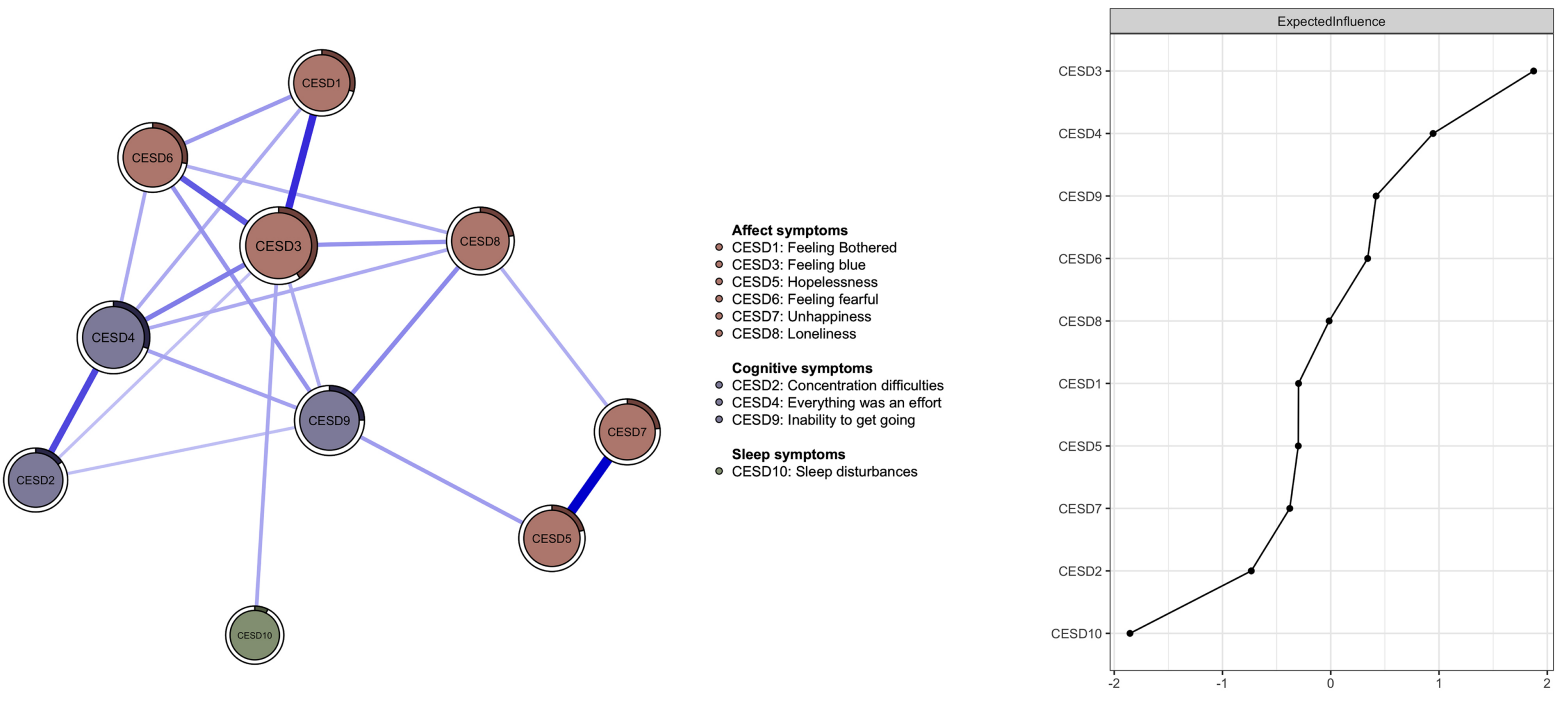
**Network structure of depressive symptoms among older adults with cataract**.

The network structure of depressive symptoms is shown in Fig. 2. The most influential node was CESD3 “Feeling blue” (EI = 1.9), followed by CESD4 “Everything was an effort” (EI = 0.9), and CESD9 “Inability to get going” (EI = 0.4) (**Supplementary Table 1**). There was significant difference in network structure between the poor economic level and the fair economic level (*p* of network invariance test = 0.917, *p* of global strength invariance test = 0.003) (**Supplementary Fig. 1**).

**Fig. 3.**
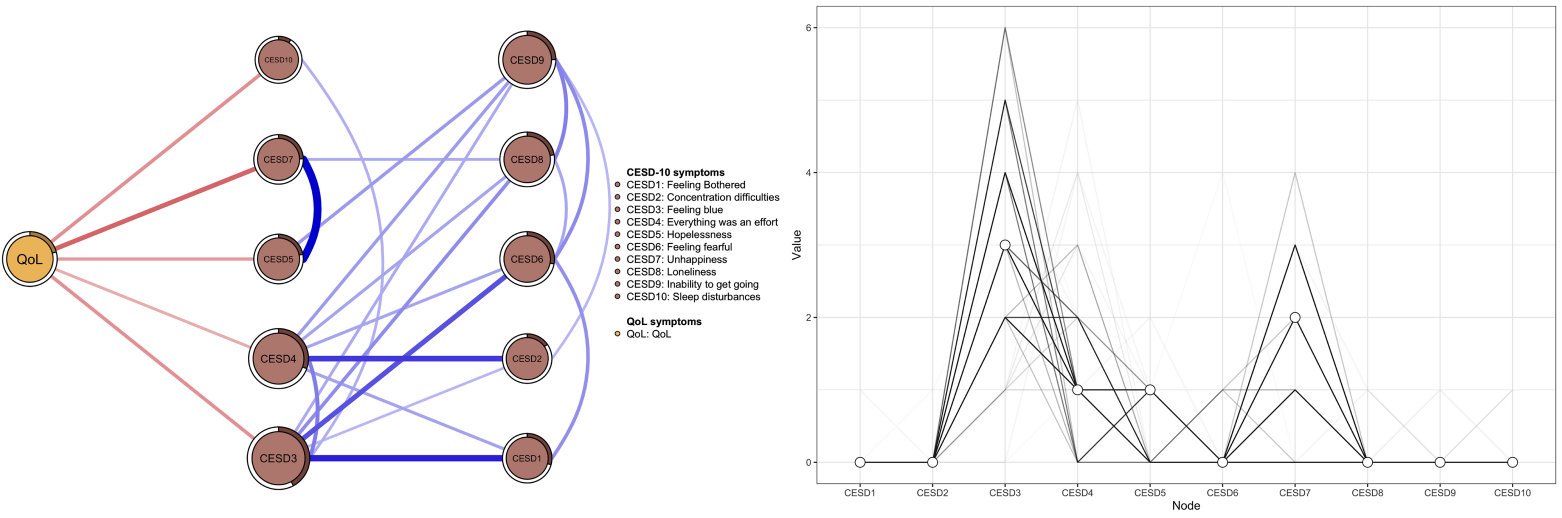
**Flow network for quality of life and depressive symptoms**.

The flow network model of QoL and depressive symptoms is shown in Fig. 3. Only CESD3 “Feeling blue”, CESD4 “Everything was an effort”, CESD5 “Hopelessness”, CESD7 “Unhappiness” and CESD10 “Sleep disturbances” were directly connected to QoL. Among those, node-specific predictive betweenness from QoL showed that the strongest mediating node between QoL and other depressive nodes was CESD3 “Feeling blue” and CESD7 “Unhappiness” (Fig. 3). Moreover, the strongest linkages were observed in edge QoL - CESD7 “Unhappiness” (edge weight = –0.185), followed by QoL - CESD10 “Sleep disturbances” (edge weight = –0.127), and QoL – CESD3 “Feeling blue” (edge weight = –0.126).

### 3.5 Network Stability of Depression With QoL

The network’s stability was assessed using the CS-coefficient for strength and EI, both of which were above 0.75, indicating high reliability (**Supplementary Fig. 2**). The accuracy of the network model was demonstrated by the narrow 95% CIs for estimated edge weights from non-parametric bootstrapping (**Supplementary Fig. 3**). Moreover, the model’s reliability was confirmed by the significant differences identified in bootstrapped edge weight tests (**Supplementary Fig. 4**). Average case-drop bootstraps of node-specific betweenness from QoL showed that overall the stability was not highly reliable, but CESD3 “Feeling blue” retained relatively high node-specific betweenness across case-drops (**Supplementary Fig. 5**).

## 4. Discussion

### 4.1 Main Findings

To the best of our knowledge, this was the first study to explore the network structure of depressive symptoms among older adults with cataracts. We found that depression was common in this population, especially in those who had a poor economic status. In addition, we found that “Feeling blue” was the most central symptom within the network model and “Unhappiness” was the most significant direct association to QoL.

Among 1683 older adults with cataracts included in this study, the prevalence of depression (CESD-10 total score ≥10) was 16.5% (95% CI: 14.5–18.3%). In comparison to a recent meta-analysis of 27 studies, the prevalence of depression was 30% among individuals with visual impairment [28]. Our finding is slightly lower than a previous study in China which found that the prevalence of depressive symptoms was 23.9% (95% CI: 19.4–28.4%) among patients with cataracts [29]. The high prevalence of depression might be associated with poor visual acuity in patients with cataracts who did not have cataract surgery [30]. Having severe cataracts was also associated with higher levels of visual disability, poorer quality of life and more severe comorbidities [31], all of which might increase the risk of depression. Similar to previous findings that poorer economic status was associated with higher risk of depression in Chinese older adults [32], we found that older adults with cataracts who had a lower economic status also had an increased risk of depression. This might be attributed to having limited access to medical services due to lack of funds [33], which could lead to worsening cataracts and increasing risk of depression. Moreover, studies have reported that financial support for medical expenses among older adults with chronic diseases, such as cataracts, could reduce the risk of depression caused by economic pressure [34]. Our study further found that economic status had no impact on the network structure of depression. However, since the sample sizes for those with poor and fair economic status were different, this result should be interpreted with caution. The biological mechanism underlying the high risk of depression associated with cataracts remains unclear. It might be mediated by D-amino acids [35], particularly D-asp, which could alter the higher-order structure of lens crystallin proteins, potentially contributing to the development of cataracts [35, 36]. Meanwhile, previous research suggested that D-amino acids and their metabolites are involved in the pathophysiology of depression through the brain–gut–microbiota axis [37]. Further basic research is needed to elucidate the possible mechanisms involved.

In the network model “Feeling blue” was identified as the most influential (central) symptom, which is consistent with previous network analyses [38]. “Feeling blue” is defined as a pervasive feeling of sadness or emotional distress associated with depression [39]. Cataracts in older adults is characterized by clouding of the lens and visual impairment, which is likely to restrict physical mobility, daily activities, independence, and social engagement in older adult [9]. Such restrictions could worsen the feelings of sadness and “feeling blue”, which in turn might lead to the development of depression [9]. On the other hand, previous research revealed that “feeling blue” might constitute a reaction to life changes such as decreased mobility caused by physical illness [40]. In addition, “Everything was an effort” and “Inability to get going” were also the other key central symptoms, both of which might relate closely to cognition, underscoring the relevance of cognitive symptoms in activating and maintaining the network model of depression among older adults with cataracts. These symptoms could also reflect having fatigue or experiencing increased burden as part of depression, which could arise from decreased mobility as a result of impaired vision due to cataracts [9, 40].

In the flow network model assessing the interplay between QoL and depressive symptoms, the most negative correlation identified with QoL was characterized as “Unhappiness”. Prior research found that a lack of happiness could be a pivotal risk factor for having suicidal ideation, poor clinical outcomes, and functional impairment [41], all of which might lower QoL. Furthermore, “Sleep disturbances” was identified as another symptom negatively associated with QoL, which is consistent with prior findings [42] that highlighted the impact of disrupted sleep patterns on QoL in those with major depressive disorder. We also found that “Feeling blue” was another symptom negatively associated with QoL. The activation of depressive symptoms characterized as “Feeling blue” might precipitate a cascade of symptoms in the depression network model, which could further lower QoL. Notably, node-specific predictive betweenness analyses revealed that “Feeling blue” served as a mediator between QoL and other depressive symptoms. This implied a potential sequential activation that decreased QoL by initially triggering “Feeling blue”, and subsequently catalyzing the onset of other depressive symptoms. Given the important role of “Feeling blue” within the network of depression, our findings suggest that interventions targeting this symptom could potentially decrease the progression of other manifestations of depression.

### 4.2 Strengths and Limitations

This study had multiple strengths, chief among them being the considerable sample size and the representativeness of the participant group, which enhanced the generalizability of the findings within the defined demographic population. Another strength was the use of novel and sophisticated statistical analyses to examine the inter-relationships between depressive symptoms. However, there were several limitations. First, the reliance on self-reported data for both depressive symptoms and QoL could introduce recall bias, thus affecting the accuracy of the data collected. Secondly, the cross-sectional nature of the study restricted the ability to infer causality between depressive symptoms and QoL. Third, the findings might not apply to populations other than older adults over 65 years with cataracts.

## 5. Conclusions

In this large-scale prospective cross-sectional study, we found that depression was common among older adults with cataracts. After controlling for risk variables, older adults with cataracts who had a lower economic status showed an increased risk of depression. To reduce the negative impact on QoL, the central symptoms (“Feeling blue”, “Everything was an effort” and “Inability to get going”) and those associated with QoL (“Unhappiness”, “Sleep disturbances” and “Feeling blue”) should be prioritized in developing appropriate interventions to address depression in this population.

## Availability of Data and Materials

The data in this study were sourced from the CLHLS and are available at https://doi.org/10.3886/ICPSR38899.v1.

## Supplementary Material

Supplementary material associated with this article can be found, in the online version, at https://doi.org/10.31083/AP45683.
