# Supplementary material for: Depressive Symptoms and Their Association With Quality of Life in Older Adults With Cataracts: A National Survey in China
Source: Alpha Psychiatry. 2025 Aug 11;26(4):45683. doi: 10.31083/AP45683 (PMC12416046; doi:10.31083/AP45683)
Supplement: Supplementary file 1 [file 2757-8038-26-4-45683-s1.docx]

**Supplementary materials**

**Supplementary Table 1.** Network descriptive information of depressive symptoms

**Supplementary Fig. 1.** Comparison of network properties between females and males

**Supplementary Fig. 2.** Estimation of Expected Influence and Strength difference on 1,000 case-dropping bootstrap samples of the flow network

**Supplementary Fig. 3.** Bootstrapped confidence intervals of edge weights on 1,000 nonparametric bootstrap samples of the flow network

**Supplementary Fig. 4.** Estimation of edge difference on 1,000 nonparametric bootstrap samples of the flow network

**Supplementary Fig. 5.** Average case-drop bootstraps of node-specific betweenness based on 1,000 iterations of the flow network

**Supplementary Table 1.** Network descriptive information of depressive symptoms

| Item | Item content | Predictability | EI |
| --- | --- | --- | --- |
| CESD1 | ‘Feeling Bothered’ | 0.3 | -0.3 |
| CESD 2 | ‘Concentration difficulties’ | 0.2 | -0.7 |
| CESD 3 | ‘Feeling blue’ | 0.4 | 1.9 |
| CESD 4 | ‘Everything was an effort’ | 0.3 | 0.9 |
| CESD 5 | ‘Hopelessness’ | 0.2 | -0.3 |
| CESD 6 | ‘Feeling fearful’ | 0.3 | 0.3 |
| CESD 7 | ‘Unhappiness’ | 0.2 | -0.4 |
| CESD 8 | ‘Loneliness’ | 0.2 | 0.0 |
| CESD 9 | ‘Inability to get going’ | 0.2 | 0.4 |
| CESD10 | ‘Sleep disturbances’ | 0.1 | -1.9 |

Note: Expected Influence based on " z-scores" to plot standardized coefficients.

**Supplementary Fig. 1.** Comparison of network properties between poor economic level and fair economic level


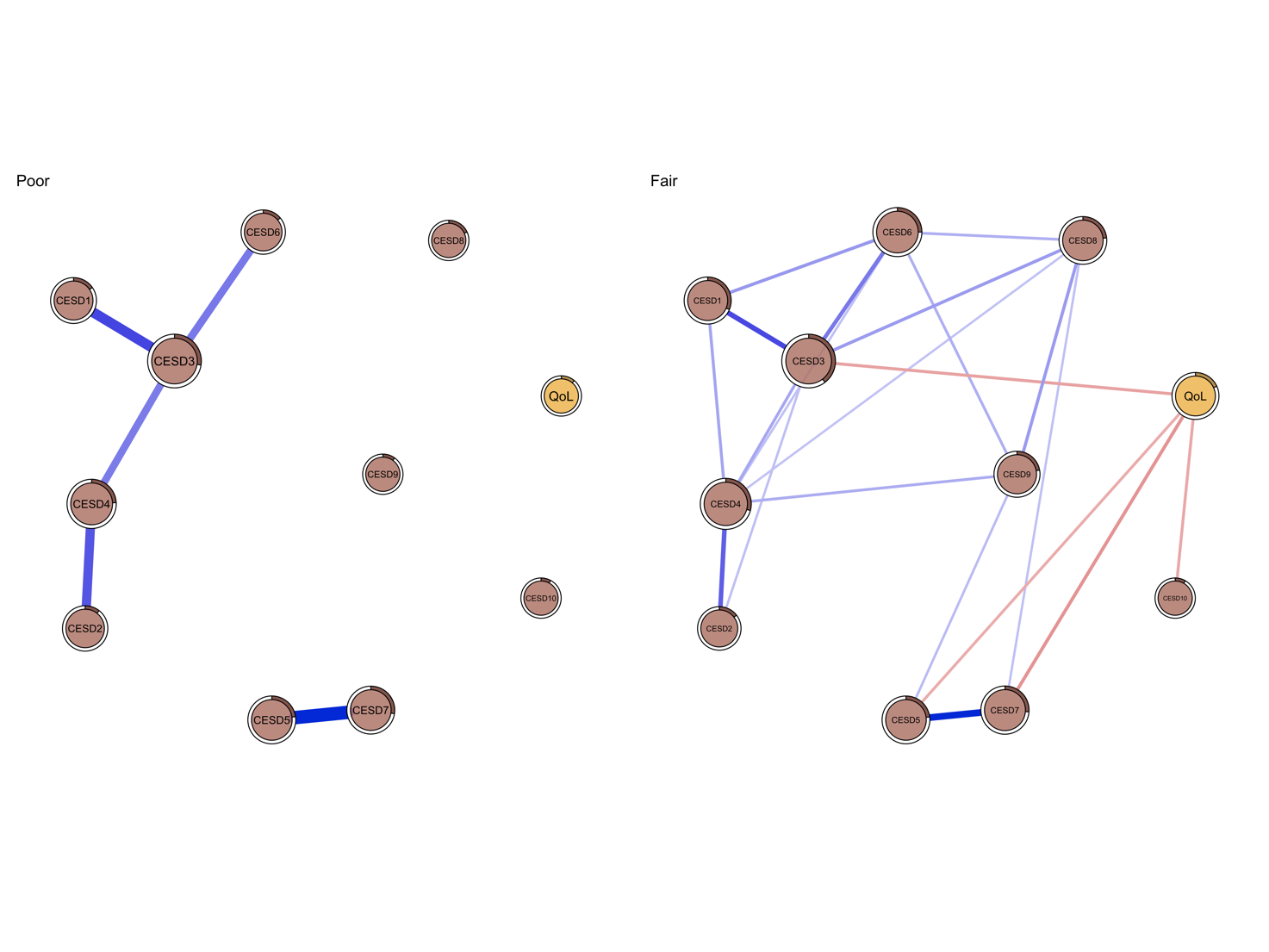


**Supplementary Fig. 2.** Stability of centrality indices on 1,000 case-dropping bootstrap samples of the flow network


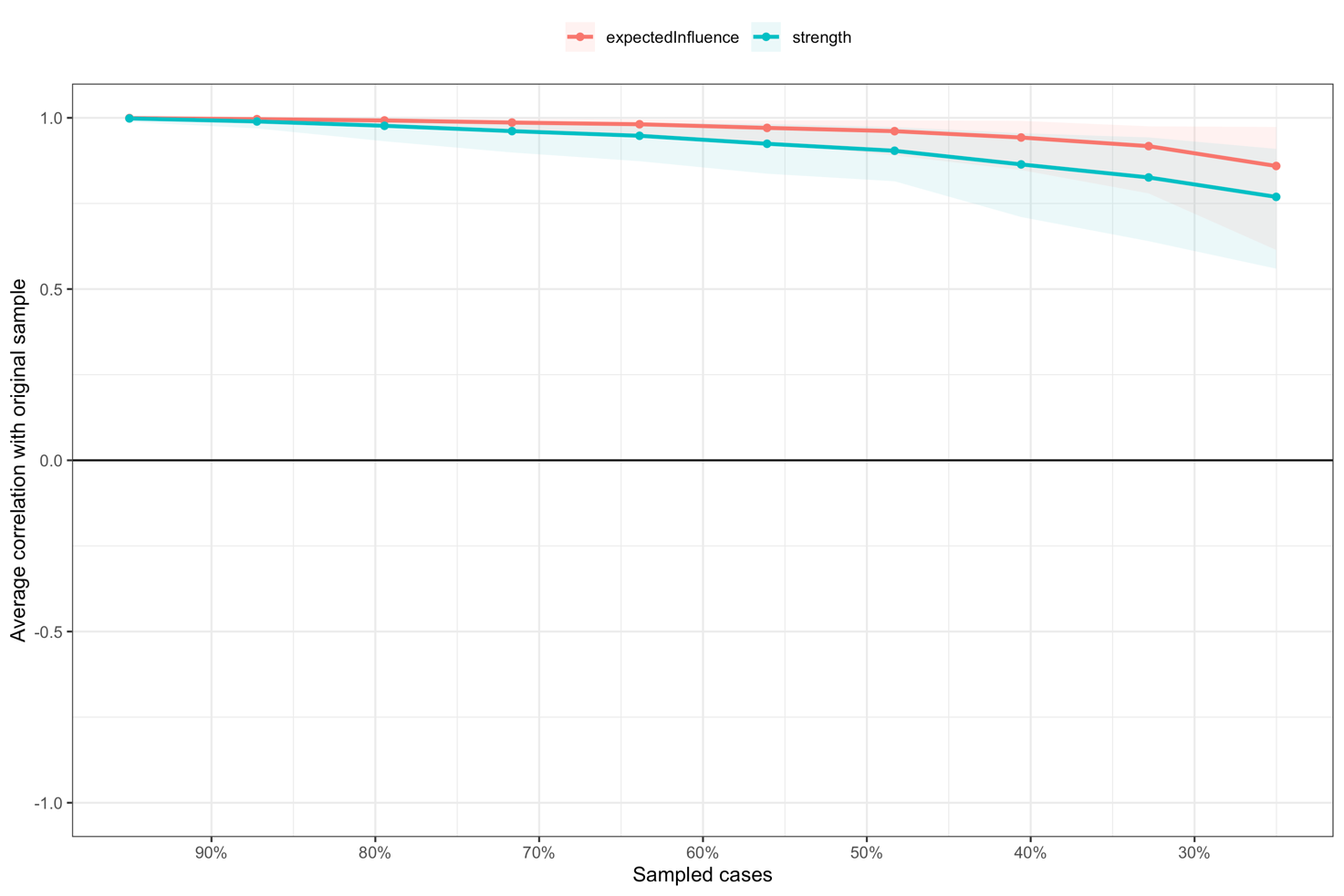


Notes: Centrality indices interpretable based on correlation stability coefficient (CS-coefficient), which over 0.75 means our network stability can be interpreted as high.

**Supplementary Fig. 3.** Bootstrapped confidence intervals of edge weights on 1,000 nonparametric bootstrap samples of the flow network


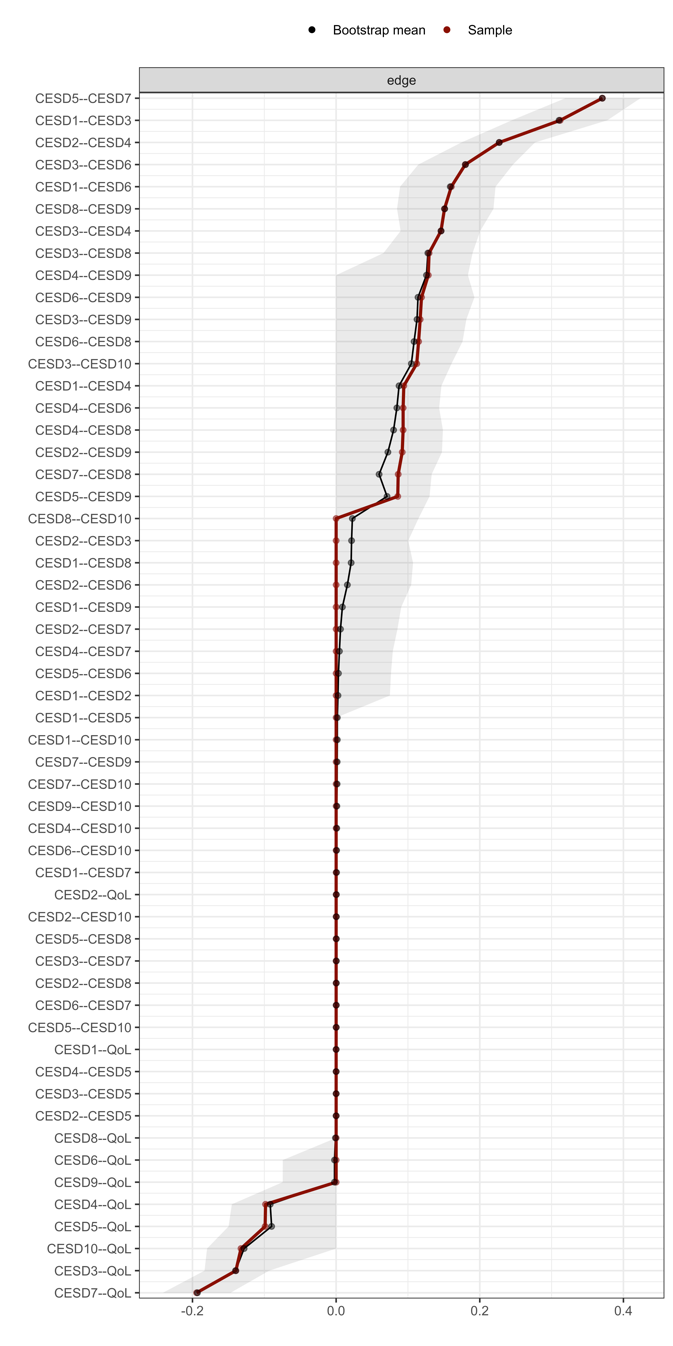


Notes: Each horizontal line indicates the 95% quantile range of the parameter values when the parameter was included in the model. The transparency of each line indicates the proportion of times the edge was included in the model (more transparent lines indicate edges that were included less often). The red dots within the plot indicate the sample values for the analyzed data, while the grey areas indicate bootstrapped confidence intervals.

**Supplementary Fig. 4.** Estimation of edge difference on 1,000 nonparametric bootstrap samples of the flow network


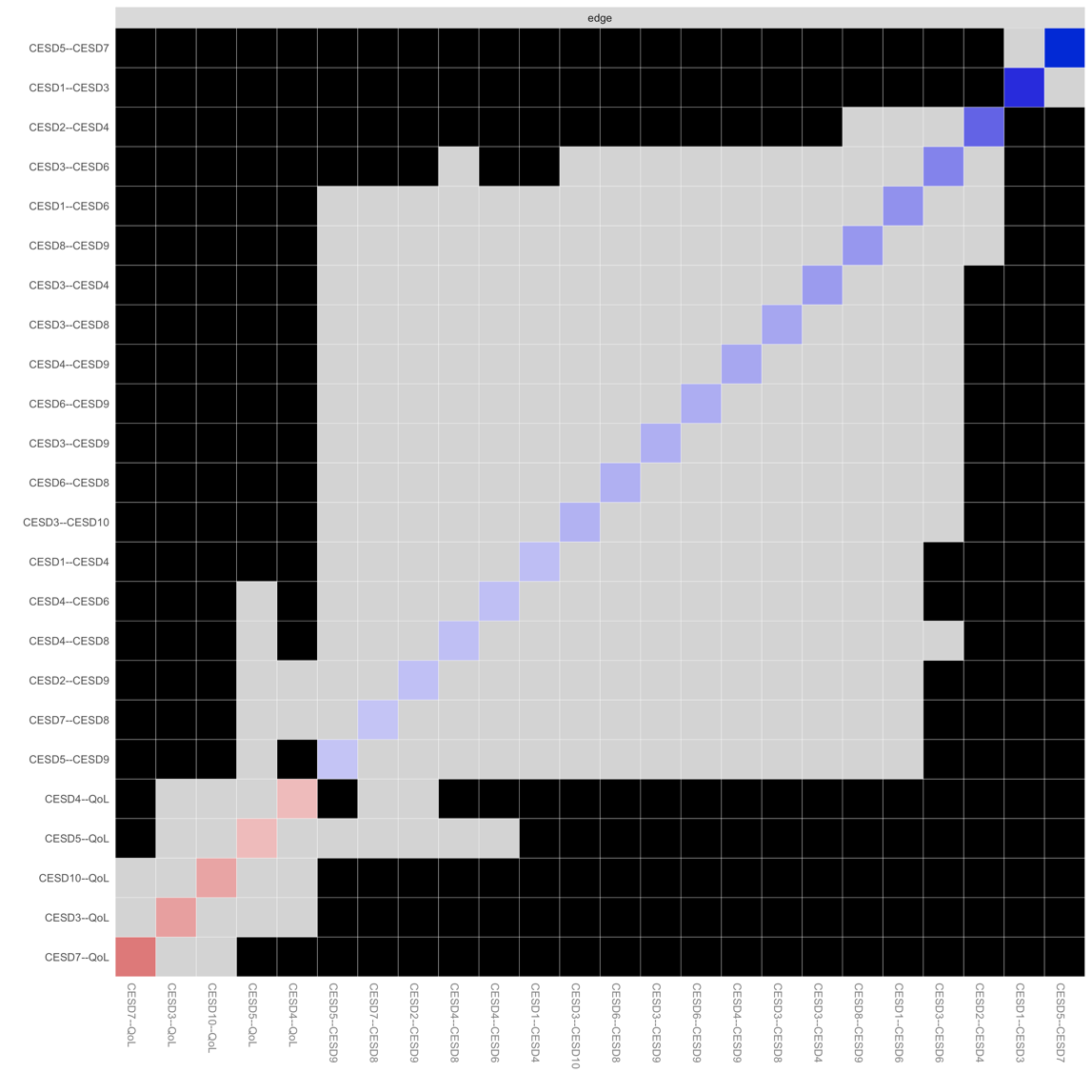


Notes: Gray boxes indicate edges that do not differ significantly from one-another and black boxes represent edges that do differ significantly from one-another. Blue boxes in the edge-weight plot indicate positive correlations. Here, we can observe many edges that significantly differ from each other, meaning the network stability can be interpreted as high.

**Supplementary Fig. 5.** Average case-drop bootstraps of node-specific betweenness, based on 1,000 iterations

~~
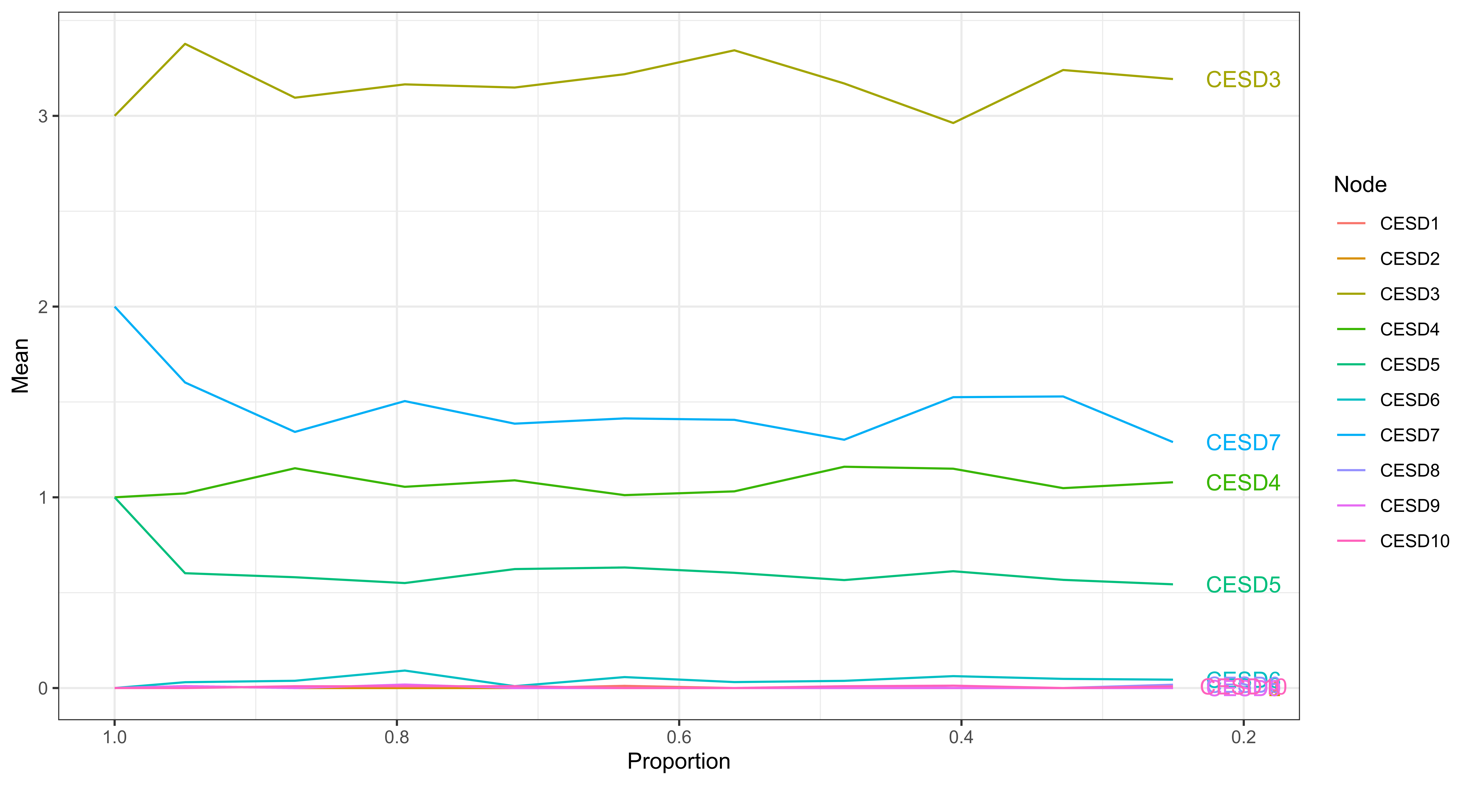
~~

The lines represent how node-specific betweenness (how often a node lies on the pathway between two other nodes, of which one is always the QoL) changes for each variable when dropping different proportions of the data.
